# Association between dietary protein intake and risk of chronic kidney disease: a systematic review and meta-analysis

**DOI:** 10.3389/fnut.2024.1408424

**Published:** 2024-06-14

**Authors:** Yu Cheng, Guanghao Zheng, Zhen Song, Gan Zhang, Xuepeng Rao, Tao Zeng

**Affiliations:** The Second Affiliated Hospital, Jiangxi Medical College, Nanchang University, Nanchang, China

**Keywords:** total protein, plant protein, animal protein, chronic kidney disease, meta-analysis

## Abstract

**Objective:**

There is suggestive data indicating a correlation among dietary protein intake and the progression of chronic kidney disease (CKD). Nonetheless, the exact associations between dietary protein intake and the incidence of CKD have remained uncertain. We performed the first meta-analysis to explore the correlation among total protein, plant protein, animal protein intake and CKD risk.

**Methods:**

The study conformed the PRISMA statement guidelines. We comprehensively searched PubMed, Web of Science, and Embase until to December 2023. The retrieved studies underwent rigorous evaluation for eligibility, and relevant data were meticulously extracted. The Newcastle-Ottawa Scale (NOS) tool was applied to evaluate the risk of bias. Subsequently, relevant data were extracted and pooled to evaluate the relations among dietary protein intake and CKD incidence.

**Results:**

Totally, 6,191 articles were identified, six studies were eligible. A total of 148,051 participants with 8,746 CKD cases were included. All studies had a low overall risk of bias. Higher total, plant and animal protein intake were all correlated with decreased CKD incidence, pooled risk ratios (RRs) and 95% confidence intervals (CIs) were as follows: (RR = 0.82, 95% CI = 0.71–0.94, *p* = 0.005; I^2^ = 38%, *p* = 0.17); (RR = 0.77, 95% CI = 0.61–0.97, *p* = 0.03; I^2^ = 77%, *p* = 0.001); (RR = 0.86, 95% CI = 0.76–0.97, *p* = 0.02; I^2^ = 0%, *p* = 0.59), respectively. For fish and seafood within animal protein: RR = 0.84, 95% CI = 0.74–0.94. Subgroup analysis showed that geographical region, sample size, follow-up time, not assessing protein by food frequency questionnaire, using %energy as the measurement index, not adjusting for several covariates may be the sources of heterogeneity for plant protein. A significant non-linear relation among plant protein and incident CKD was observed by dose–response analysis.

**Conclusion:**

The data showed a lower CKD risk significantly associated higher-level dietary total, plant or animal protein (especially for fish and seafood) intake. Further prospective studies demonstrating the correlations of precise sources, intake and duration of dietary protein and incident CKD are warranted.

## Introduction

Chronic kidney disease (CKD) causes an elevated occurrence of many conditions involving multiple organs and systems and premature mortality. Furthermore, a considerable number of those with CKD could progress to renal failure, necessitating hemodialysis (1, 2). Given that CKD is a chronically progressive and nonreversible condition, primary prevention becomes imperative, even without kidney damage.

Previous studies showed that dietary interventional measures could be effective in slowing the progression of the disease and reducing associated complications (3). About 90% of protein metabolic waste are excreted by the kidneys. Consequently, a higher protein intake may result in glomerular hyperfiltration and damage the kidney (4, 5). Hence, although the clinical evidence of benefit was limited, some guidelines recommend dietary interventional measures. The 2012 Kidney Disease: Improving Global Outcomes (KDIGO) guideline suggested maintaining a protein intake of 0.8 g protein/kg (weight)/d for those with diabetes and CKD not treated with dialysis (6). Additionally, for CKD patients not on dialysis and without diabetes, in adults with CKD 3–5 who are metabolically stable, the National Kidney Foundation’s Kidney Disease Outcomes Quality Initiative (KDOQI) recommended under close clinical supervision, protein restriction with or without keto acid analogs, to reduce risk for end-stage kidney disease (ESKD) and improve quality of life (7). Nevertheless, these guidelines did not provide recommendations of specific sources, as well as the advices of dietary protein intake for the general population.

Previous researchers have focused on the relationship among dietary protein intake and kidney function in population without CKD. A meta-analysis indicated that high-level protein diets were correlated with growing estimated glomerular filtration rate (eGFR) among individuals without CKD (8). However, studies that explored the association between dietary protein intake as well as specific sources and incident CKD were scarce. Two studies reported a beneficial correlation between high-level total protein intake and incident CKD (9, 10), while three studies showed no significant relation of that (11–13). Of those, four studies indicated a beneficial correlation between high-level plant protein intake and incident CKD (10–12, 14), while one study showed no significant correlation (9). In addition, three studies suggested no correlation among high-level animal protein intake and incident CKD (10–12), while one study showed beneficial correlation (9). Therefore, the relation between dietary protein intake as well as specific sources and the incidence of CKD remains uncertain.

With this background, we performed the first systematic review and meta-analysis to determine the exact correlations of dietary protein intake and CKD incidence.

## Materials and methods

### Data sources, literature search, and study selection

The study conformed the PRISMA statement guidelines (15). The researchers performed a detailed retrieval of potential eligible studies from PubMed/Medline, Web of Science, and Embase from the inception of these databases up until December 25, 2023, using the following search terms: (protein [Title]) AND [(chronic kidney disease [Title/Abstract]) OR (CKD [Title/Abstract])]. We focused on studies written in the English language that involved human research. Furthermore, the authors manually searched the reference lists of eligible articles.

Two reviewers (YC, TZ) examined the titles and abstracts of potentially relevant articles. If there was disagreement, a consensus was achieved by discussing the issue and seeking input from a third reviewer (GH Z). The included articles met the following criteria: (1) explored the relationship among dietary protein intake and incident CKD, (2) was a prospective or case–control study, (3) reported outcome indicators such as hazard ratios (HR), odds ratios (OR), relative risks (RR) and 95% confidence interval (CI), or provided sufficient data to calculate them, (4) performed the age-adjustment. Exclusion criteria: (1) explored the relationship among dietary protein intake and CKD progression rather than incident CKD, (2) retrospective studies, (3) did not report outcome indicators, (4) did not adjust age.

### Data extraction and risk of bias assessment

The detailed data of first author, year of publication, country, study name and design, sample description (sample size, age, sex), follow-up time, risk estimates (95% CI), and adjustment variables were extracted and the risk of bias was evaluated independently for the enrolled articles by two reviewers (YC and TZ). In cases where there was disagreement, decision was made by a third reviewer (GH Z).

The Newcastle-Ottawa Scale (NOS) for cohort and case–control studies was used to assessed the risk of bias of the included studies (16). The scale consists of eight questions that cover three aspects: (1) Selection (up to four stars); (2) Comparability (up to two stars); and (3) Exposure (for case–control studies, up to three stars) or Outcome (for cohort studies, up to three stars). An overall risk of bias was categorized as: Low (7–9 stars), Medium (4–6 stars), or High (<4 stars).

### Statistical analysis

The HRs and ORs were considered as the RRs. The standard errors (se) was calculated as (log(upper bound 95% CI of RR) – log(RR)) /1.96. The log(RR)s were weighted by w_i_, calculated as 1/(se^2^ + t^2^), where t^2^ represented the restricted maximum likelihood estimate of the overall variance (17). We referred to the Cochrane Handbook for Systematic Reviews of Interventions (18) as a guide to perform the meta-analysis. In all analyses, we selected the risk estimate that was adjusted to its maximum extent. To evaluate heterogeneity, we used Q- and I^2^-statistics (17). However, publication bias analysis was not conducted due to insufficient studies available (19).

Subgroup analyses were performed by stratifying geographic region (Asian and Western), sample size (<7,000 and ≥ 7,000), age (average or median: <55 and ≥ 55), follow up years (<10 and ≥ 10), whether to assess protein intake by food frequency questionnaire (FFQ), and whether to adjust for race, carbohydrate intake, low-density lipoprotein cholesterol (LDL), triglycerides (TG), systolic blood pressure (SBP), fasting blood sugar (FBS). Sensitivity analyses were carried out by systematically excluding individual studies to assess the impact of each study on the overall results. This evaluation aimed to determine the robustness of the findings when any single study was omitted. Additionally, a dose–response analysis was conducted for plant protein. However, due to lack of data, we cannot perform a similar dose–response analysis for animal protein.

RevMan software version 5.4 and R software version 4.3.2 were used. A *p* value less than 0.05 was statistically significant.

## Results

### Study selection

**Figure 1:**
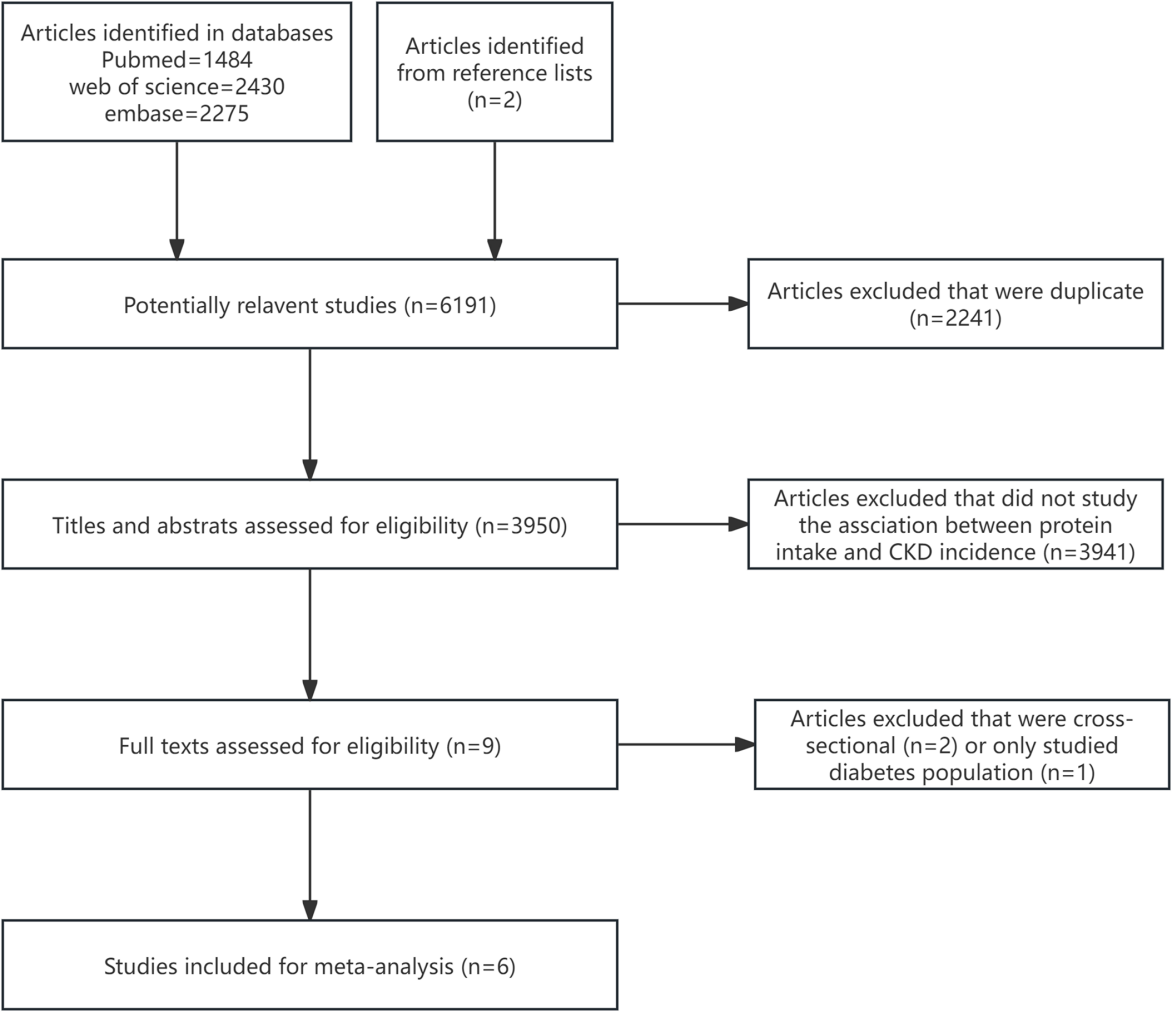
Flow diagram of the literature search strategy for the meta-analysis.

Totally, 6,191 publications were identified, 6,189 records from databases and two records from reference lists (Figure 1). Titles and abstracts of 3,950 studies were assessed by removing duplicate studies. Full-text of nine studies were evaluated. Two studies were cross-sectional and one study only included diabetes population. Finally, six studies were enrolled (9–14).

### Study characteristics

All studies were based on prospective cohort design. Four studies derived from Asian countries and two studies derived from the US and UK. Totally, 148,051 participants and 8,746 CKD cases were included. Four studies adopted FFQ to evaluate dietary protein intake, one study used 24-h recall questionnaire and another study used brief-type self-administered diet history questionnaire (BDHQ). Five studies reported risk estimates of HR and one study reported OR. Five studies investigated the correlation among total protein or plant protein intake and CKD risk, and four studies explored animal protein intake. The detailed data was presented in Table 1. A low overall risk of bias were observed in these six studies (Table 2).

**Table 1:** Characteristics of included studies.

First author (year)	Country	Study design	Population	Follow up	Protein intake (assessment, outcome for high vs. low)	Adjustment variables
Alvirdizadeh (2020)	Iran	TLGS study, PC	Size: 1630 Age: 42.8 years Sex: male, female	6.1 years	FFQ, OR (95% CI) Total protein: 0.59 (0.32–1.08) Plant protein: 0.28 (0.14–0.53) Animal protein: 0.91 (0.57–1.44)	Age, sex, BMI, smoking, TEI, PA, total fiber intake and energy percent from fat, diabetes, hypertension, FBS and SBP
Haring (2018)	USA	ARIC study, PC	Size: 11952 Age: 53.8 years Sex: male, female	23 years	FFQ, HR (95% CI) Total protein: 0.89 (0.76–1.05) Plant protein: 0.76 (0.64–0.91) Animal protein: 0.91 (0.78–1.06)	Age, race, sex, BMI, education, alcohol, smoking, TEI, TCI, HDL, LDL, TG, TCHO, LLDs, SBP, AHDs, PA, leisure-time PA, and WHR
Heo (2023)	England	UK Biobank, PC	Size: 117809 Age: 55.3 years Sex: male, female	9.9 years	24-h recall questionnaire HR (95% CI) Plant protein: 0.82 (0.73–0.93)	Age, race, sex, BMI, socioeconomic status, alcohol, smoking, PA, TEI, fat, protein, carbohydrate, and sodium, hypertension, diabetes, CVD, chronic pulmonary disease, and liver disease, RAAS blockers, diuretics, statins, eGFR, UACR, LDL, TG, and hs-CRP
Kubo (2023)	Japan	CIRCS, PC	Size: 3277 Age: 58.8 years Sex: male, female	8.1 years	BDHQ, HR (95% CI) Total protein: 0.72 (0.52–0.99) Plant protein: 1.24 (0.89–1.75) Animal protein: 0.77 (0.56–1.08)	Age, sex, community, BMI, smoking, alcohol, DBP, AHDs, diabetes, TCHO, LLDs, TEI, eGFR
Kwon (2022)	Korea	KoGES, PC	Size: 7339 Age: 51.8 years Sex: male, female	13.7 years	FFQ, HR (95% CI) Total protein: 0.63 (0.44–0.89) Plant protein: 0.72 (0.54–0.95) Animal protein: 0.74 (0.55–1.00)	Age, sex, obesity, PA, smoking, alcohol, protein intake per total energy intake, and phosphorus intake, SBP, FBS, LDL, and CRP
Teymoori (2022)	Iran	TLGS study, PC	Size: 6044 Age: 37.9 years Sex: male, female	7.7 years	FFQ, HR (95% CI) Total protein: 0.91 (0.78–1.05)	Age, sex, BMI, smoking, PA, education, TEI, baseline GFR, SBP, FBS, TC and sodium

PC, prospective cohort; FFQ, food frequency questionnaire; BDHQ, brief-type self-administered diet history questionnaire; OR, odds ratio; HR, hazard ratio; CI, confidence interval; BMI, body mass index; TEI, total energy intake; PA, physical activity; FBS, fasting blood sugar; SBP, systolic pressure; TCI, total carbohydrate intake; HDL, high-density lipoprotein; LDL, low-density lipoprotein; TG, triglycerides; TCHO, total cholesterol; LLDs, lipid-lowering drugs; AHDs, anti-hypertension drugs; WHR, waist-to-hip ratio; CVD, cardiovascular disease; RAAS, renin-angiotensin-aldosterone system; eGFR, estimated glomerular filtration rate; UACR, urinary albumin-creatinine ratio; hs-CRP, high-sensitivity C-reactive protein.

**Table 2:** Risk of bias assessment of included studies.

Study	Selection (up to 4 stars)	Comparability (up to 2 stars)	Outcome (up to 3 stars)	Overall bias
Alvirdizadeh 2020	****	**	**	Low
Haring 2018	***	**	***	Low
Heo 2023	***	**	***	Low
Kubo 2023	***	**	**	Low
Kwon 2022	****	**	**	Low
Teymoori 2022	****	**	**	Low

Overall risk of bias: Low (7–9 stars); Medium (4–6 stars); High (<4 stars).

### Dietary protein intake and CKD risk

**Figure 2:**
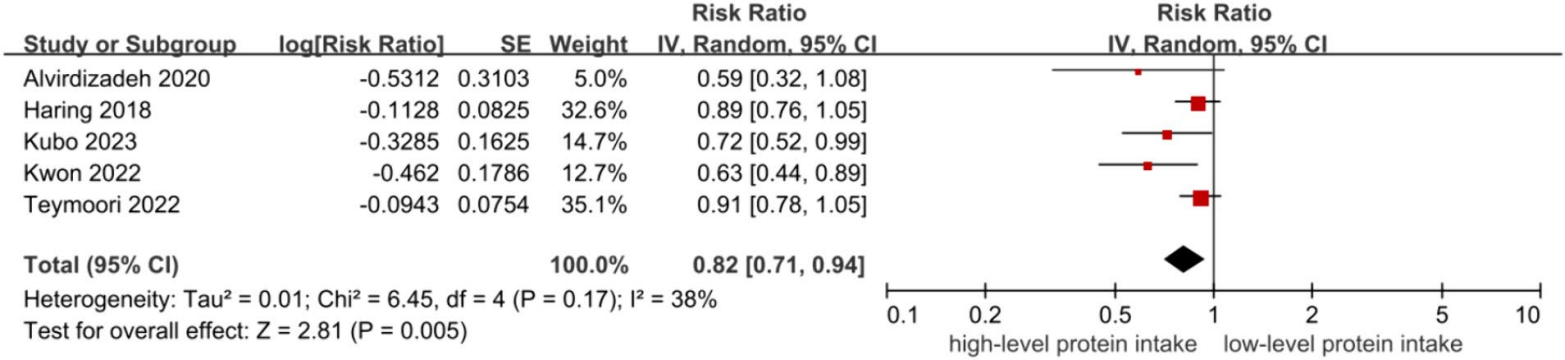
Total protein and risk of CKD.

**Figure 3:**
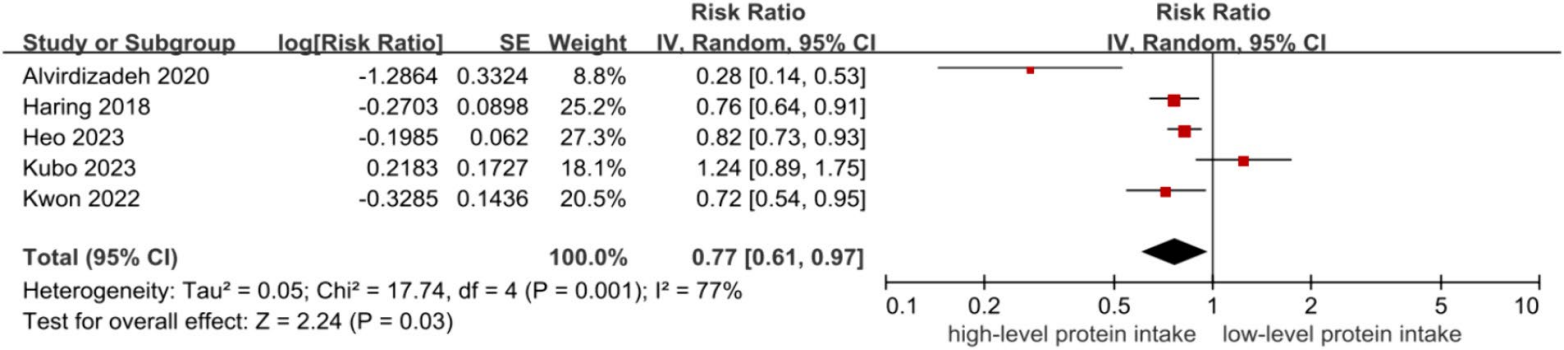
Plant protein and risk of CKD.

**Figure 4:**
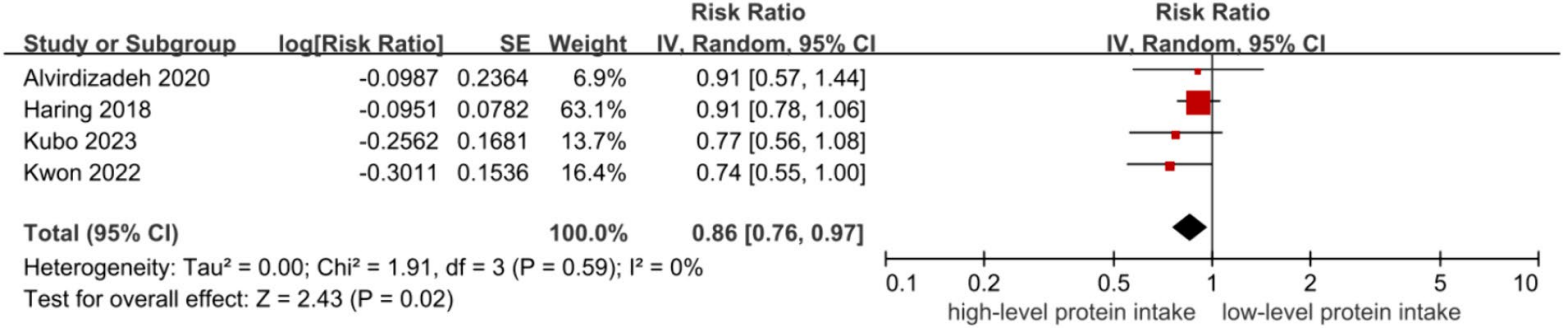
Animal protein and risk of CKD.

The inverse associations between higher level of protein intake and risk of CKD were all observed in dietary total protein (RR = 0.82, 95% CI = 0.71–0.94, Figure 2) (9–13), plant protein (RR = 0.77, 95% CI = 0.61–0.97, Figure 3) (9–12, 14) and animal protein intake (RR = 0.86, 95% CI = 0.76–0.97, Figure 4) (9–12), with a random-effect model. For animal protein, the pooled data of fish and seafood was (RR = 0.84, 95% CI = 0.74–0.94) (9, 12). There was low heterogeneity among the studies for total protein (I^2^ = 38%, P-heterogeneity = 0.17) and animal protein (I^2^ = 0%, P-heterogeneity = 0.59), while plant protein had a significantly high heterogeneity (I^2^ = 77%, P-heterogeneity = 0.001).

### Subgroup analysis

Due to a significantly high heterogeneity of plant protein, we performed the subgroup analysis to explore the sources of heterogeneity. The results showed that the correlations among plant protein intake and CKD incidence were statistically significant without significant heterogeneity among studies that derived from Western countries (RR = 0.80, 95% CI = 0.72–0.89; I^2^ = 0%, *p* = 0.51), had a sample size ≥7,000 (RR = 0.79, 95% CI = 0.72–0.87; I^2^ = 0%, *p* = 0.63), had a follow-up time ≥ 10 years (RR = 0.75, 95% CI = 0.65–0.87; I^2^ = 0%, *p* = 0.73), adjusted for race (RR = 0.80, 95% CI = 0.72–0.89; I^2^ = 0%, *p* = 0.51), adjusted for carbohydrate (RR = 0.80, 95% CI = 0.72–0.89; I^2^ = 0%, *p* = 0.51), adjusted for LDL cholesterol (RR = 0.79, 95% CI = 0.72–0.87; I^2^ = 0%, *p* = 0.63) and adjusted for TG (RR = 0.80, 95% CI = 0.72–0.89; I^2^ = 0%, *p* = 0.51), which indicated that these factors may be the sources of high heterogeneity for plant protein (Table 3).

**Table 3:** Subgroup analysis of plant protein intake.

Subgroups	Number of studies	RR [95% CI]	*I* ^2^	*p*-value for *I*^2^
Total	5	0.77 [0.61–0.97]	77%	0.001
Geographic region				
Western countries	2	0.80 [0.72–0.89]	0%	0.51
Asian countries	3	0.67 [0.34–1.29]	88%	<0.001
Sample size				
<7,000	2	0.60 [0.14–2.63]	94%	<0.001
≥7,000	3	0.79 [0.72–0.87]	0%	0.63
Age				
<55	3	0.61 [0.42–0.89]	77%	0.01
≥55	2	0.98 [0.65–1.47]	81%	0.02
Follow up time				
<10 years	3	0.72 [0.42–1.24]	88%	<0.001
≥10 years	2	0.75 [0.65–0.87]	0%	0.73
Assessed by FFQ				
Yes	3	0.61 [0.42–0.89]	77%	0.01
No	2	0.98 [0.65–1.47]	81%	0.02
Adjusted for race				
Yes	2	0.80 [0.72–0.89]	0%	0.51
No	3	0.67 [0.34–1.29]	88%	<0.001
Adjusted for carbohydrate				
Yes	2	0.80 [0.72–0.89]	0%	0.51
No	3	0.67 [0.34–1.29]	88%	<0.001
Adjusted for LDL				
Yes	3	0.79 [0.72–0.87]	0%	0.63
No	2	0.60 [0.14–2.63]	94%	<0.001
Adjusted for TG				
Yes	2	0.80 [0.72–0.89]	0%	0.51
No	3	0.67 [0.34–1.29]	88%	<0.001
Adjusted for SBP				
Yes	3	0.61 [0.42–0.89]	77%	0.01
No	2	0.98 [0.65–1.47]	81%	0.02
Adjusted for FBS				
Yes	2	0.47 [0.18–1.19]	86%	0.008
No	3	0.87 [0.71–1.06]	69%	0.04

RR, risk ratio; CI, confidence interval; FFQ, food frequency questionnaire; LDL, low-density lipoprotein cholesterol; TG, triglycerides; SBP, systolic blood pressure; FBS, fasting blood sugar.

### Sensitivity analysis

Sensitivity analysis showed that the results were stable for total and animal protein by deleting a single study at a time. However, for plant protein, CKD incidence became non-statistically significant by omitting Haring et al.’s (12), Heo et al.’s (14) or Kwon et al.’s (10) study. However, the weights of these three studies were the highest of all, which indicated the importance to the pooled results. In addition, I^2^ of the heterogeneity decreased from 77% (*p* = 0.001) to 59% (*p* = 0.06) by removing Alvirdizadeh et al.’s study with a risk estimates of OR.

### Dose–response meta-analysis

**Figure 5:**
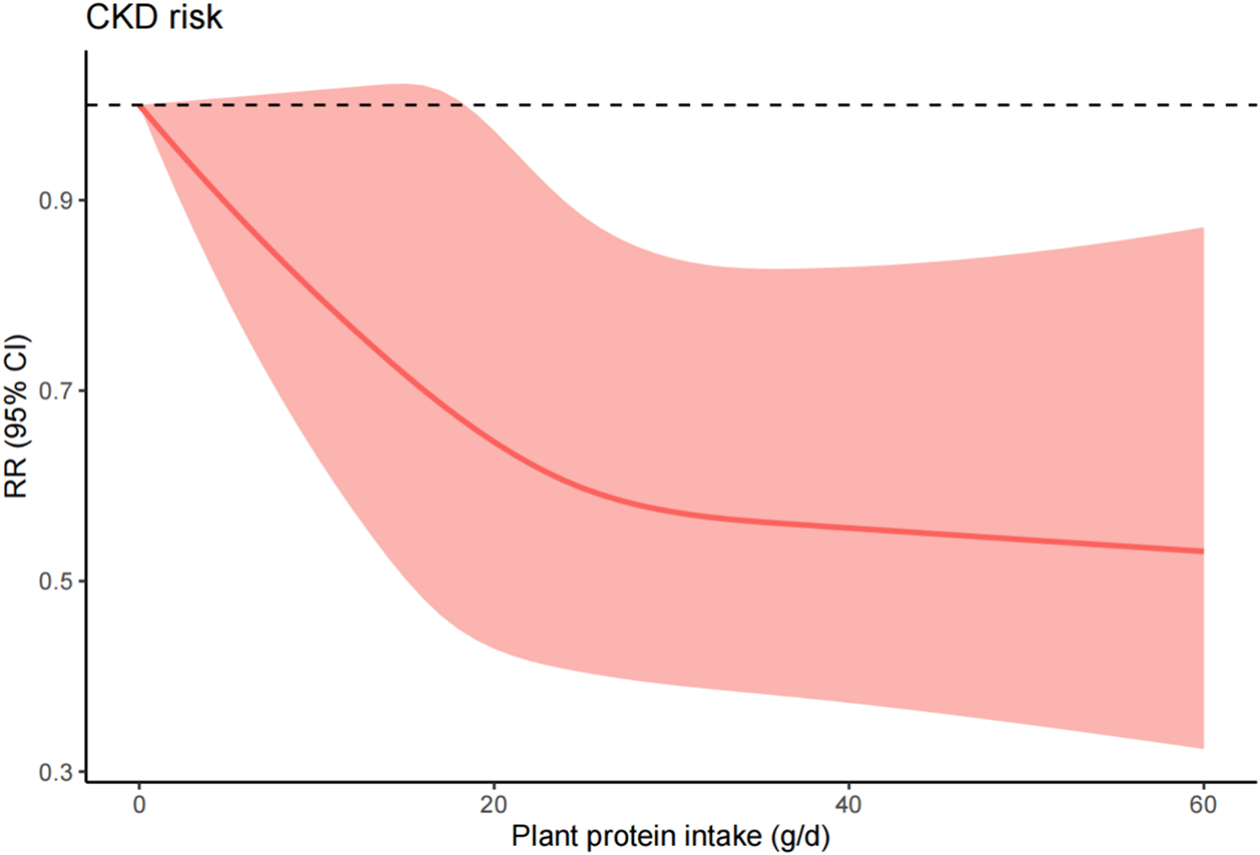
Dose response analysis quantifying the association between plant protein intake and CKD. Analysis includes all studies reporting on three or more physical activity categories.

Due to the lack of enough data for total protein and animal protein, we only conducted the dose–response meta-analysis for plant protein (12, 14). The dose response meta-analysis revealed a non-linear relation between plant protein intake and CKD incidence (*p* < 0.001). The intake of 15 g/d, 30 g/d, and 45 g/d for plant protein were associated with reductions in CKD risk of 28% (RR = 0.72, 95% CI = 0.50–1.02), 43% (RR = 0.57, 95% CI = 0.39–0.84), and 45% (RR = 0.55, 95% CI = 0.36–0.84), respectively (Figure 5).

## Discussion

Our data showed that a higher-level intake of total, plant and animal protein can lower a risk of CKD by 18, 23 and 14%, respectively. Specifically, a high-level of fish and seafood with animal protein can lower a 16% risk of CKD. A significant non-linear correlation was found among plant protein intake and incident CKD by dose–response analysis. All studies were prospective cohort studies with a low overall risk of bias.

Current studies’ conclusions remain controversial in terms of the correlation between total protein intake and CKD risk. Other than these studies included in this meta-analysis, a cross-sectional study reported that there was no significant association between total protein intake and incident CKD, involving 5,316 participants aged 27 years or older without diabetes (20). In another cross-sectional study conducted in Japan, high-level total protein intake associated with reduced CKD risk was found in women but not in men, and a beneficial relationship was observed between total protein intake and eGFR in both men and women (21). However, due to the cross-sectional nature, these two studies cannot be pooled in the present meta-analysis. Moreover, a randomized clinical trial revealed that increased total protein intake from 91.4 to 107.8 g/day can increase the level of eGFR and the volume of kidney among healthy overweight or obese men and women (22). Indeed, evidence suggested that the beneficial effect of total protein intake in persons without kidney insufficiency could be attributed to protein-induced hyperfiltration, wherein the kidneys adapt to an increased BUN and Scr (23).

Studies have focused on the preventive effect of various dietary patterns on chronic conditions. “Healthy foods” like vegetables and fruits are typically recommended, whereas there have been concerns raised regarding red and processed meat (24–26). An ARIC Study involving 11,952 adults with an eGFR of ≥60 mL/min/1.73 m^2^ found that higher consumption of red meat and processed meat can significantly increase the incidence of CKD G3. Conversely, foods rich in vegetable protein can significantly reduce the occurrence of CKD (12). Another study conducted in Iran, which included 4,881 participants, found that substituting red or processed meat with foods rich in vegetable protein correlated with a reduced incidence of CKD (27). Further studies also demonstrated that higher plant protein intake was associated with a lower risk of subsequent CKD (11, 20). Similarly, a study involving 7,339 middle-aged and older Korean adults also confirmed that (10). Notably, a recent prospective study with a 9.9-median year follow-up of a large sample size of 117,809 participants from UK biobank revealed that greater dietary plant protein intake was associated with a lower risk of incident CKD (14). However, we found only one study reported no significant correlation between vegetable protein intake and CKD incidence (9).

There is compelling evidence supporting the benefit for plant proteins in promoting kidney health. More pronounced inflammation will be induced by a high-level animal protein intake rather than vegetable protein intake. For instance, the inflammatory macrophage responding and a series of cytokines releasing were observed in a colitis mice model fed with a diet rich in animal proteins rather than plant proteins (28). Moreover, consuming animal proteins significantly burdens the kidneys (29). Diets high in vegetables and low in animal protein exhibit higher proportions of glutamic acid, cystine, proline, phenylalanine, and serine compared to diets low in vegetables and high in animal protein (30). These differences in amino acid content likely led to distinct nitrogen loads and acidogenicity levels (31, 32). It’s worth noting that plant-based foods are also notably rich in dietary fiber, which plays a crucial role in altering gut microbiota composition, lowering circulating cholesterol levels, mitigating inflammation (33–36), and reducing the occurrence of CKD (37). Taken together, the mounting evidence substantiated a clear association among a high-level vegetable protein and diminished incident CKD.

Interestingly, our meta-analysis also suggested a beneficial correlation among a high-level animal protein intake and incident CKD. Caution must be exercised when interpreting this finding. Previous studies revealed that high-level of red meat and processed meat intake significantly increased the incidence of CKD (12, 27, 38). In the contrary, fish and seafood were reported an inverse correlation with CKD incidence (9, 12, 27). Two studies included in animal protein analysis derived from Japan and Korea in which fish and seafood were main consumption of animal protein (39, 40), this may explain the inverse relation among high animal protein intake and lower risk of CKD in this meta-analysis. Mechanistically, the protective effect of fish and seafood may be attributed to an anti-inflammatory effect of long-chain n-3 polyunsaturated fatty acids from seafood (41). However, after removal of these two studies, the beneficial effect of high-level animal protein intake was not statistically significant.

We found no significant heterogeneity among studies within total protein and animal protein intake, but these studies focused on plant protein presented a significant high heterogeneity. Subgroup analysis showed that the heterogeneity was non-statistically significant among studies that derived from Western countries, had a sample size ≥7,000, had a follow-up time ≥ 10 years, adjusted for race, adjusted for carbohydrate, adjusted for LDL cholesterol and adjusted for TG, which suggested the potential sources of heterogeneity. Sensitivity analysis showed that Alvirdizadeh et al.’s study (11) may be part of the sources of heterogeneity in plant protein. This study reported a risk estimate of OR, while others reported HRs that considered time factor.

Our meta-analysis has several strengths. One is that it was the first meta-analysis paying attention to the effect of high-level protein intake on CKD incidence. In addition, subgroup analysis can identify potential sources of heterogeneity. Importantly, the included studies were all prospective cohort studies with a low overall risk of bias, which provided a methodologic rigor to allow these results to be interpreted with high confidence.

However, there are some limitations to our meta-analysis. Firstly, the number studies on this topic was still small. Secondly, a significant high heterogeneity was observed in studies within plant protein intake. Thirdly, the definitions of CKD were inconsistent. Four studies adopted eGFR<60 mL/min/1.73 m^2^ as the diagnostic criteria (9–11, 13), while one study used records (14) and another study considered CKD stage 3 as the outcome (12). Lastly, the measurement indexes of protein intake were not standardized. Two studies used %energy of protein intake as the indicator (9, 13), while four studies used absolute intake as the indicator (10–12, 14). In addition, the criteria of categories were also inconsistent, although a dose–response meta-analysis was performed to determine the relation of the detailed intake and CKD risk, the data was still limited. These factors may cause bias to the results. Future studies should standardize the criteria of CKD diagnosis, measurement indicator and categorizing protein intake. Additionally, studies should also focus on the precise sources, detailed intake and duration of dietary protein relevant for potential decreased risk of CKD. In addition, studies should be focused more on subgroup population as well as the population with different disease such as obesity, hypertension and diabetes.

## Conclusion

This study showed that a high-level intake of dietary total protein, plant protein and animal protein intake (especially for fish and seafood) can significantly reduce 18, 23 and 14% CKD risk. Future studies should focus on the specific sources, detailed intake and duration of dietary protein relevant for potential decreased risk of CKD.

## Data availability statement

The original contributions presented in the study are included in the article/supplementary material, further inquiries can be directed to the corresponding author.

## Author contributions

YC: Conceptualization, Data curation, Formal analysis, Investigation, Methodology, Project administration, Resources, Software, Supervision, Visualization, Writing – original draft, Writing – review & editing. GuZ: Data curation, Methodology, Software, Writing – original draft, Writing – review & editing. ZS: Formal analysis, Supervision, Validation, Writing – original draft, Writing – review & editing. GaZ: Data curation, Formal analysis, Writing – original draft, Writing – review & editing. XR: Data curation, Investigation, Writing – original draft, Writing – review & editing. TZ: Conceptualization, Data curation, Formal analysis, Investigation, Methodology, Project administration, Resources, Software, Supervision, Validation, Visualization, Writing – original draft, Writing – review & editing.
